# Effect of *Salvia leriifolia *Benth. root extracts on ischemia-reperfusion in rat skeletal muscle

**DOI:** 10.1186/1472-6882-7-23

**Published:** 2007-07-07

**Authors:** Hossein Hosseinzadeh, Azar Hosseini, Marjan Nassiri-Asl, Hamid-Reza Sadeghnia

**Affiliations:** 1 Department of Pharmacology and Toxicology, Pharmaceutical Research Center, Faculty of Pharmacy, Mashhad University of Medical Sciences, Mashhad, I.R. Iran; 2 Faculty of Medicine, Mashhad University of Medical Sciences, Mashhad, I. R. Iran; 3 Department of Pharmacology, Faculty of Medicine, Qazvin University of Medical Sciences, Qazvin, I.R. Iran; 4 Department of Pharmacology, School of Medicine, Mashhad University of Medical Sciences, Mashhad, I.R. Iran

## Abstract

**Background:**

*Salvia leriifolia *have been shown to decrease ischemia-reperfusion (I/R) injury in brain tissues. In this study, the effects of *S. leriifolia *aqueous and ethanolic extracts were evaluated on an animal model of I/R injury in the rat hind limb.

**Methods:**

Ischemia was induced using free-flap surgery in skeletal muscle. The aqueous and ethanolic extracts of *S. leriifolia *(100, 200 and 400 mg/kg) root and normal saline (10 ml/kg) were administered intraperitoneally 1 h prior reperfusion. During preischemia, ischemia and reperfusion conditions the electromyographic (EMG) potentials in the muscles were recorded. The markers of oxidative stress including thiobarbituric acid reactive substances (TBARS), total sulfhydryl (SH) groups and antioxidant capacity of muscle (using FRAP assay) were measured.

**Results:**

In peripheral ischemia, the average peak-to-peak amplitude during ischemic-reperfusion was found to be significantly larger in extracts groups in comparison with control group. Following extracts administration, the total SH contents and antioxidant capacity were elevated in muscle flap. The MDA level was also declined significantly in test groups.

**Conclusion:**

It is concluded that *S. leriifolia *root extracts have some protective effects on different markers of oxidative damage in muscle tissue injury caused by lower limb ischemia-reperfusion.

## Background

A vast amount of circumstantial evidence implicates oxygen-derived free radicals (especially superoxide and hydroxyl radical) and high-energy oxidants (such as peroxynitrite) are as mediators of inflammation, shock, and ischemia/reperfusion injury [1]. The oxidant injury can potentially occur during ischemia and reperfusion due to an excess production of oxygen free radicals, a decrease in antioxidant defenses, or both. Because antioxidants function by removing the toxic oxygen metabolites, they are generally highly effective in reducing ischemia-reperfusion injury [2]. Skeletal muscle ischemia and reperfusion injury remains an issue of concern because of the morbidity and mortality that follows revascularization of an acutely ischemic limb [3]. Many studies have suggested the beneficial antioxidant effects of antioxidant nutrients such as vitamin E, green tea extract, ginkgo biloba extract, resveratrol and niacin in cerebral ischemia and recirculation brain injury [4].

The plants of the genus *Salvia*, which consist about 900 species [5] are generally known for their multiple pharmacological effects such as analgesic and anti-inflammatory [6], antioxidant [7], hepatoprotective [8] and hypoglycemic activities [9]. *Salvia leriifolia *that was introduced in flora Iranica in 1982 [10] geographically grows in the south and tropical regions of Khorassan and Semnan provinces, I.R. Iran. In recent years, the different pharmacological activities of this plant such as the attenuation of morphine dependence [11], hypoglycemic [12], analgesic and anti-inflammatory [13,14], anticonvulsant [15], antiulcer effects [16] and antibacterial activities [17] were evaluated in our laboratory.

The anti-ischemia effects of some species of *Salvia *such as *S. hematodes *and *S. miltiorrhiza *[18,19] have been reported. Our studies also showed the anti-ischemia effects of *S. leriifolia *extracts in hippocampus of rats [20]. Thus, in this study the effect of *S. leriifolia *extract was evaluated during ischemia-reperfusion on an animal model of I/R injury in the rat hind limb.

## Methods

### Animals

Wistar male rats, 200–230 g were housed in colony rooms with 12/12 h light/dark cycle at 21 ± 2°C and had free access to food and water. All animal experiments were carried out in accordance with Mashhad University of Medical Sciences, Ethical Committee Acts.

### Chemicals

TBA, n- butanol, phosphoric acid, potassium chloride and thiobarbituric acid were purchased from Merck. Xylazine and ketamine were obtained from Loughrea, Co. (Galway, Ireland) and Rotexmedica (GmbH, Germany), respectively. All chemical were dissolved in distilled water.

### Induction of ischemia

The rats were anesthetized with intraperitoneal injection of ketamine/xylazine (60 mg/kg and 6 mg/kg, respectively). Additional doses of these agents were used if anesthesia lightened during experiment.

An incision in the inner side of the hind leg from the inguinal ligament to the tendon calcaneus insertion was made. Then it was divided up and the triceps surae was dissected as a muscle flap, after that insertions to femur was cut. Previously dissected femoral vessels, the artery and vein were clamped with a single clamp of microsurgery. The absence of bleeding was verified in the muscle flap. Then the incision was closed to prevent desiccation. For reperfusion periods, the clamp of the femoral vessels of animals was taken off and then the bleeding of the muscle flap was verified. The muscle tissues was homogenized in cold KCl solution (1.5%) to give a 10% homogeny suspension and used for biochemical assays. The results were expressed by n or μM/g tissue (1 ml of homogenate = 0.1 g of tissue) [49].

Eight groups of animals were used, each of which contained 8 rats: Group 1 including sham operated animals, Group 2 served as ischemic control to which saline (10 ml/kg) was injected intraperitoneally (i.p.). Group 3–8 was received the aqueous and ethanolic extract (100, 200 and 400 mg/kg). Except group 1, other groups underwent 2 h ischemia and 1 h reperfusion. All drugs were administrated 1 h before reperfusion.

### Electromyography data collection

To determine the muscles activities during ischemia-reperfusion, intramuscular electromyograph (EMG) signals were recorded with PowerLab data acquisition systems. Two pairs of pin electrodes in terminating alligator clips were inserted into the triceps surae (muscle flap) in hind leg, and adductor muscles. The distance between the two electrodes of a pair in each muscle was 5 mm. A grounding electrode was gently attached to the rat tail [50]. The EMG signals were collected with sampling frequency of 12 PPM (MacLab/4SP). Duration for each stimulation was 20 ms. The raw EMG signals were low-pass filtered at 50 Hz and EMG signal is expressed as average peak-to-peak amplitude for a 10 min recording periods. The electromyography (EMG) signals were recorded 10 min before ischemia, 10 min before reperfusion and 10 min at end of reperfusion phase.

### Preparation of Salvia leriifolia extracts

*S. leriifolia *was collected from Bajestan (Razavi Khorasan province, Northeast of Iran) and authenticated by Pharmacognosy Department, School of Pharmacy, MUMS, Iran (Voucher No:153-1912-05). For preparation of aqueous extract, the powdered root (100 g) was boiled in 1000 ml boiling water for 15 min. Subsequently, the mixture was filtered and concentrated under reduced pressure at 35°C (yield: 5.5% w/w). As some constituents are sensitive to boiling water we also prepared a macerated extract. For preparation of the ethanolic extract, the powdered root (100 g) was macerated in 1000 ml ethanol (96% v/v) for 72 h and subsequently the mixture was filtered and concentrated under reduced pressure at 35°C (yield: 6% w/w).

#### Preliminary chemical tests

Phytochemical screening of the extract was performed using the following reagents and chemicals (Trease and Evans, 1983): alkaloids with Dragendorff's reagent, flavonoids with the use of Mg and HC1.; tannins with 1 % gelatin and 10% NaCl solutions and saponins with ability to produce suds and hemolysis reaction [51].

#### Characterization of aqueous and ethanolic extracts by HPLC

The quality of extracts of *S. leriifolia *radix was characterized by HPLC finger print. The aqueous and ethanolic extracts were dissolved in distilled water and acetonitrile respectively and then filtered through 0.22 μm membrane filter. Twenty μl of sample (10 g/l) was injected to the reverse phase column (C18). The mobile phase was consisted water: phosphoric acid 1N, 99:1 (as solvent A) and acetonitrile: phosphoric acid 1N, 99:1 (as solvent B) with a gradient elution (0, 95:5; 5, 92.5:7.5; 10, 90:10; 15, 87.5:12.5; 20, 85:15; 25, 82.5:17.5; 30, 80:20; 35, 78.5:22.5, 40, 75:25) at the flow rate of 1 ml/minute. The peaks were monitored at 236 nm.

### Thiobarbituric acid reactive substances (TBARS) measurement

Malondialdehyde (MDA) levels, as an index of lipid peroxidation, were measured. MDA reacts with thiobarbituric acid (TBA) as a thiobarbituric acid reactive substance (TBARS) to produce a red colored complex which has peak absorbance at 532 nm [52].

3 ml phosphoric acid (1%) and 1 ml TBA (0.6%) was added to 0.5 ml of homogenate in a centrifuge tube and the mixture was heated for 45 min in a boiling water bath. After cooling, 4 ml of n-butanol was added the mixture and vortex-mixed for 1 min followed by centrifugation at 20000 rpm for 20 min. The organic layer was transferred to a fresh tube and its absorbance was measured at 532 nm. The standard curve of MDA was constructed over the concentration range of 0–40 μM [53].

### Ferric Reducing/Antioxidant Power (FRAP) assay

The FRAP assay measures the change in absorbance at 593 nm owing to the formation of a blue colored Fe^2+^-tripyridyltriazine compound from the colorless oxidized Fe^3+ ^form by the action of electron donating antioxidants [54].

The FRAP reagent consist of 300 mM acetate buffer (3.1 g sodium acetate + 16 ml glacial acetic acid, made up to 1 liter with distilled water; pH = 3.6), 10 mM TPTZ in 40 mM HCl and 20 mM FeCl_3_.6H_2_O in the ratio of 10:1:1.

Briefly, 50 μl of muscle homogenate was added to 1.5 ml freshly prepared and prewarmed (37°C) FRAP reagent in a test tube and incubated at 37°C for 10 min. The absorbance of the blue colored complex was read against reagent blank (1.5 ml FRAP reagent + 50 μl distilled water) at 593 nm. Standard solutions of Fe^2+ ^in the range of 100 to 1000 mM were prepared from ferrous sulphate (FeSO_4_.7H_2_O) in distilled water. The data was expressed as mM ferric ions reduced to ferrous form per liter (FRAP value) [55].

### Total sulfhydryl (SH) groups assay

Total SH groups were measured using DTNB (2, 2'-dinitro-5, 5'-dithiodibenzoic acid) as the reagent. This reagent reacts with the SH groups to produce a yellow colored complex which has a peak absorbance at 412 nm [56].

Briefly, 1 ml Tris-EDTA buffer (pH = 8.6) was added to 50 μl muscle homogenate in 2 ml cuvettes and sample absorbance was read at 412 nm against Tris-EDTA buffer alone (A_1_). Then 20 μl DTNB reagent (10 mM in methanol) was added to the mixture and after 15 min (stored in laboratory temperature) the sample absorbance was read again (A_2_). The absorbance of DTNB reagent was also read as a blank (B). Total thiol concentration (mM) was calculated from the following equation:

Total thiol concentration (mM) = (A_2_-A_1_-B) × 1.07/0.05 × 13.6

### Statistical analysis

Data are expressed as mean ± SEM. Statistical analysis was performed using one-way ANOVA followed by Tukey-Kramer *post-hoc *test for multiple comparisons. The p-values less than 0.05 were considered to be statistically significant.

## Results

### Chemical analysis

**Figure 1:**
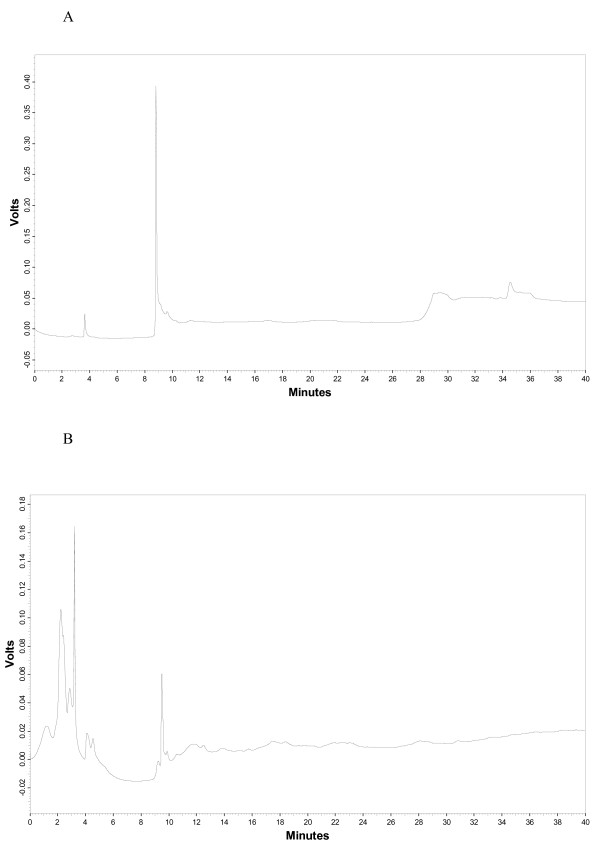
HPLC fingerprint of the ethanolic (A) and aqueous (B) extracts of *Salvia leriifolia *root.

Preliminary phytochemical tests indicated that the aqueous and ethanolic extracts of *S. leriifolia *radix contain tannins and saponins. Alkaloids were found in low amount in the ethanolic extract. HPLC fingerprints of aqueous and ethanolic extracts indicated a relatively different pattern. (Fig. 1A and 1B).

### Electromyography data

The average peak-to-peak amplitudes in the control-ischemic group and sham were 1.314 ± 0.027, 1.495 ± 0.025 V and 2.671 ± 0.018, 2.588 ± 0.033 V, p < 0.001) respectively during ischemia and reperfusion. The aqueous and ethanolic extracts increased the amplitude compare with ischemia and reperfusion groups (Table 1).

**Table 1:** Effects of *Salvia leriifolia *root aqueous and ethanolic extracts on average peak-to-peak amplitudes of EMG signals, prior, during and after ischemia-reperfusion in rat skeletal muscle.

Group (n = 8)	Preischemia (v)	Ischemia (v)	Reperfusion (v)
Sham	2.590 ± 0.015	2.671 ± 0.018***	2.588 ± 0.033***
Control group (10 ml/kg)	2.800 ± 0.044	1.314 ± 0.027	1.495 ± 0.025
Aqueous extract 100 mg/kg	2.038 ± 0.023	2.110 ± 0.030***	1.867 ± 0.067**
Aqueous extract 200 mg/kg	2.214 ± 0.036	2.296 ± 0.016***	2.303 ± 0.034***
Aqueous extract 400 mg/kg	2.440 ± 0.030	2.425 ± 0.013***	2.112 ± 0.025***
Ethanolic extract 100 mg/kg	2.040 ± 0.027	2.098 ± 0.033***	1.846 ± 0.075**
Ethanolic extract 200 mg/kg	2.190 ± 0.039	2.291 ± 0.019***	2.308 ± 0.041***
Ethanolic extract 400 mg/kg	2.430 ± 0.035	2.422 ± 0.014***	2.098 ± 0.024***

Values are mean ± S.E.M. ** P < 0.01 and ***P < 0.001 when compared with control group with Tukey-Kramer test.

### Thiobarbituric acid reactive species (TBARS) measurement

**Figure 2:**
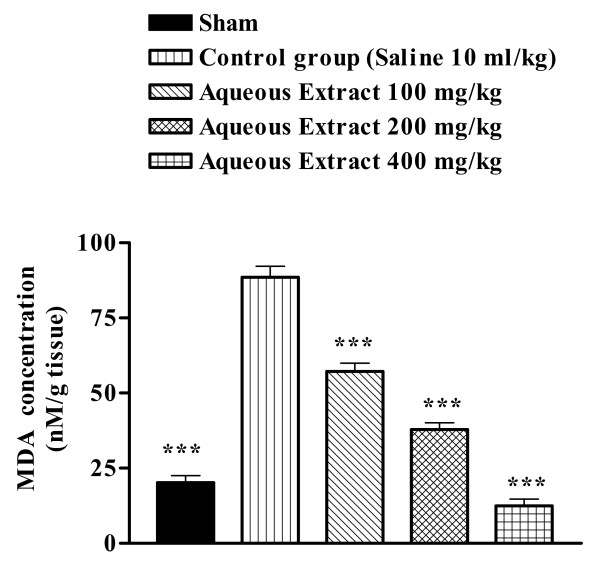
Effect of *Salvia leriifolia *root aqueous extract on lipid peroxidation following muscle ischemia reperfusion injury.MDA levels were measured in 10% homogenates of muscle samples from rats subjected to 120 min of ischemia and 60 min of reperfusion. All drugs were administrated intraperitoneally 60 min prior to reperfusion. Values are mean ± SEM (n = 8). **p < 0.01, ***p < 0.001 as compared with vehicle (normal saline) treated animals (One-way ANOVA followed by Tukey-Kramer test)

**Figure 3:**
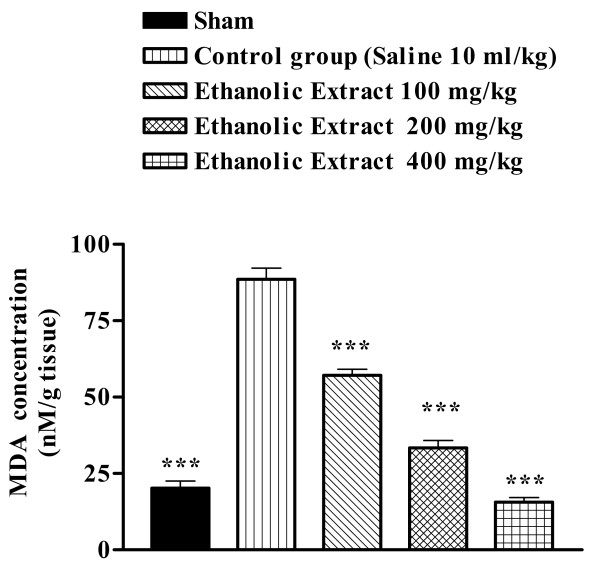
Effect of *Salvia leriifolia *root ethanolic extract on lipid peroxidation following muscle ischemia reperfusion injury. MDA levels were measured in 10% homogenates of muscle samples from rats subjected to 120 min of ischemia and 60 min of reperfusion. All drugs were administrated intraperitoneally 60 min prior to reperfusion. Values are mean ± SEM (n = 8). **p < 0.01, ***p < 0.001 as compared with vehicle (normal saline) treated animals (One-way ANOVA followed by Tukey-Kramer test)

There was an increase in the MDA levels following ischemia reperfusion as compared with sham-operated animals (20.18 ± 3.65 vs 88.53 ± 3.65 nM/g tissue, p < 0.01) (Figure 2 and 3). The extracts pretreatment resulted in a significant reduction in the free radical-mediated lipid peroxidation as indicated by a decrease in the MDA levels, at various dose levels (Figures 2 and 3). A reduction in TBARS levels was very prominent in the higher doses.

### Modulation of FRAP value

**Figure 4:**
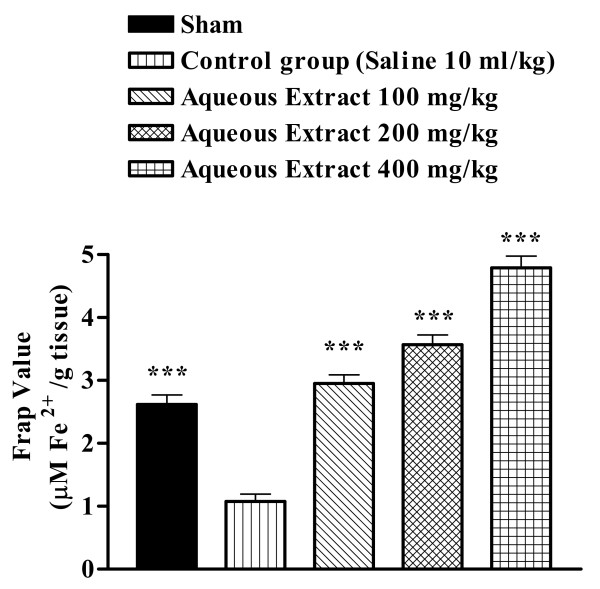
Effect of *Salvia leriifolia *root aqueous extract on antioxidant power of muscle homogenate samples following muscle ischemia reperfusion injury. FRAP values were measured in 10% homogenate samples from rats subjected to 120 min of ischemia and 60 min of reperfusion. All drugs were administrated intraperitoneally 60 min prior to reperfusion. Values are mean ± SEM (n = 8). ***p < 0.001 as compared with vehicle (normal saline) treated animals (One-way ANOVA followed by Tukey-Kramer test)

**Figure 5:**
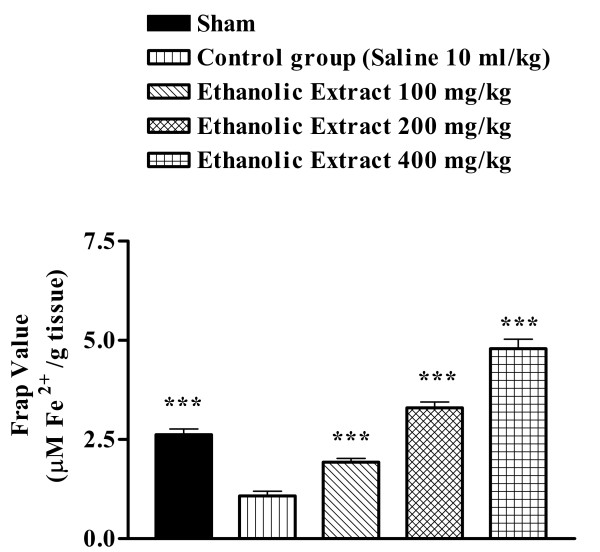
Effect of *Salvia leriifolia *root ethanolic extract on antioxidant power of muscle homogenate samples following muscle ischemia reperfusion injury. FRAP values were measured in 10% homogenate samples from rats subjected to 120 min of ischemia and 60 min of reperfusion. All drugs were administrated intraperitoneally 60 min prior to reperfusion. Values are mean ± SEM (n = 8). **p < 0.01, ***p < 0.001 as compared with vehicle (normal saline) treated animals (One-way ANOVA followed by Tukey-Kramer test)

Ischemia reperfusion caused a significant reduction in FRAP value of muscle homogenate samples as compared with sham-operated animals (2.620 ± 0.148 vs. 1.076 ± 0.119 μM/g tissue, p < 0.001) (Figure 4 and 5). The extracts pretreatment increased antioxidant power (FRAP value) of muscle homogenate samples (Figures 4 and 5).

### Total thiol concentration

**Figure 6:**
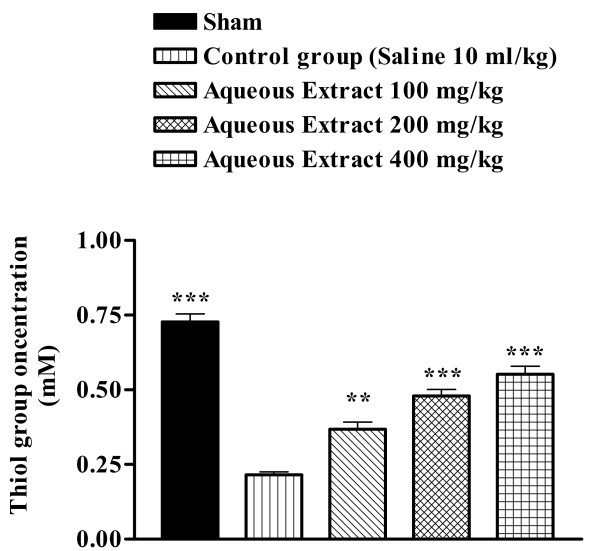
Effect of *Salvia leriifolia *root aqueous extract on total thiol concentrations following following muscle ischemia reperfusion injury. Total sulfhydryl (SH) groups were measured in 10% muscle homogenate samples from rats subjected to 120 min of ischemia and 60 min of reperfusion. All drugs were administrated intraperitoneally 60 min prior to reperfusion. Values are mean ± SEM (n = 8). ***p < 0.01 as compared with vehicle (normal saline) treated animals (One-way ANOVA followed by Tukey-Kramer test).

**Figure 7:**
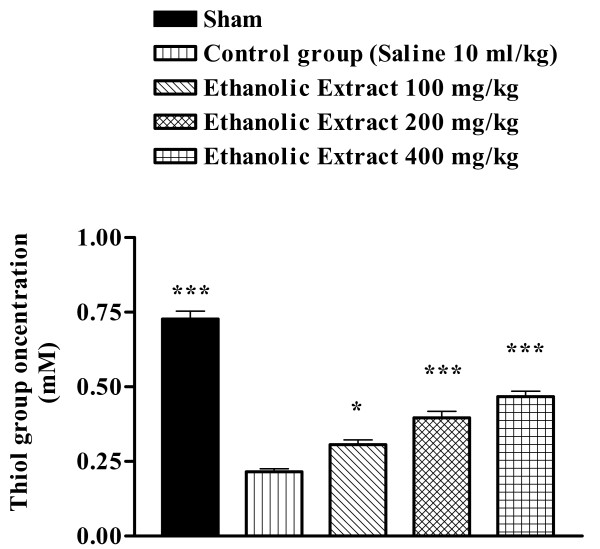
Effect of *Salvia leriifolia *root ethanolic extract on total thiol concentrations following muscle ischemia reperfusion injury. Total sulfhydryl (SH) groups were measured in 10% muscle homogenate samples from rats subjected to 120 min of ischemia and 60 min of reperfusion. All drugs were administrated intraperitoneally 60 min prior to reperfusion. Values are mean ± SEM (n = 8). *p < 0.05, ***p < 0.01 as compared with vehicle (normal saline) treated animals (One-way ANOVA followed by Tukey-Kramer test).

Following ischemia-reperfusion injury, a significant reduction in total SH groups (0.728 ± 0.027 vs. 0.215 ± 0.010 mM, p < 0.001) in muscle homogenate samples was observed (Figure 7). The extracts pretreatment caused a significant elevation in total thiol concentration, as compared with control-ischemic group (Figures 6 and 7).

## Discussion

The results obtained in the present investigation suggest that the *S. leriifolia *extracts have an overall protective effect against muscle I/R injury in a rat model.

A number of processes have been implicated in the pathogenesis of oxygen deprivation-induced cell injury. These include the disturbances of cell calcium homeostasis, depletion of adenine nucleotides, activation of enzymes like phospholipases with production of toxic lipid metabolites, proteases and endonucleases and generation of free radicals (ROS) that can cause oxidative damage to cellular macromolecules [21,22]. Antioxidant therapy has been well documented to help in the improvement of organ functions [23].

We assessed the effect of the *S. leriifolia *extracts studying their effects on lipid peroxidation, which was measured in terms of MDA, a stable metabolite of the free radical-mediated lipid peroxidation cascade. MDA levels increased significantly following muscle ischemia reperfusion. The *S. leriifolia *extracts reversed the increase of MDA levels to a considerable extent, thereby confirming its antioxidant role in I/R.

Sulfhydryl (SH) groups are highly-reactive constituents of protein molecules, and they participate in important biochemical and metabolic process such as cell division, blood coagulation, maintenance of protein systems and enzymatic activation including antioxidant enzymes (catalase, superoxide dismutase, etc.) [24]. There are also important scavengers of oxygen-derived free radicals [25]. SH groups known to be sensitive to oxidative damage and depleted following ischemic insult [26], therefore we studied the effect of the aqueous and ethanolic extracts of *S. leriifolia *on total thiol concentration during muscle ischemia reperfusion. Similarly, in our studies, total sulfhydryl groups were decreased following I/R injury. These agents exhibited higher SH contents than their respective controls, indicating that helped in replenishing the total thiol pool.

Under acute and chronic pathologic conditions such as ischemia, the balance between oxidant and antioxidant systems has been interrupted [27,28]. Therefore we evaluate the antioxidant or reducing potential of muscle homogenate samples following muscle ischemia reperfusion, using FRAP assay. As expected following this, a significant reduction in antioxidant power, as indicated by FRAP value, was observed. The aqueous and ethanolic extracts increased the antioxidant power of muscle homogenate samples.

*The S. leriifolia *extracts showed anti-ischemia activity in this study. Phytochemical test showed that the extracts comprised saponins and tannins. Antihypoxic and anti-ischemic activities of some of these components such as protopanaxatriol and ginsenosides have been reported [29-31]. Monoterpenes as the one of the constituents of the essential oil of *S. leriifolia *showed good antioxidant activity [32]. There are several reports about the antioxidant activity and anti-inflammatory effects of some monoterpenoids such as α-pinene. Moreover, there have been shown monoterpenoids such as terpineol and linalool have depressant effects on central nervous system, in vivo [32] and linalool competitively inhibits glutamate receptors [33]. However, the chemical constituents responsible for the pharmacological activities of the extracts remain to be investigated. Phytochemical assays and HPLC finger prints showed a little difference between these two extracts but it seems this variation does not affect on anti-ischemia effects. The maximum effect was shown with higher doses of both extracts

It seems that ischemia reperfusion injury reduced the conductivity and viability of muscles and nerves. In this study, the *S. leriifolia *extracts maintained the nerve conductivity. Nerve conduction is decreased during ischemia-reperfusion [34,35]. Mild muscle necrotic changes occur after 2–3 h ischemia [36,37]. Oxidative stress and the production of oxygen free radicals during ischemia-reperfusion is one mechanism of ischemic fiber degeneration, causing a breakdown of the blood-nerve barrier, endoneurial edema and lipid peroxidation [38]. *S. leriifolia *extracts prevented lipid peroxidation and showed antioxidant activity in this study. These effects and other activities such as anti-inflammatory effect [13,14] may preserve viability of nerve conductivity.

Present study showed that *S. lerrifolia *extracts suppressed the increase of MDA levels in the rat skeletal muscle and therefore inhibited lipid peroxidation following ischemia-reperfusion injury. The inhibition of lipid peroxidation and anti-ischemic effects are probably related to the antioxidant properties and free radical scavengering of the *S. leriifolia *extracts.

A number of study have shown that the other species of *Salvia *genus, especially *S. miltiorrihiza*, possess antioxidant activities [7,39], vasodilatory effects [40], the inhibition of nitric oxide production [41], glutamate release [42] and elevation of ATP levels (and probably adenosine) in brain [43].

Several studies have demonstrate that adenosine and its analogues possess antihypoxic, anti-ischemic [44-46] and neuromodulatory properties [47] and prevent lipid peroxidation following ischemia-reperfusion episodes [48]. It may be possible that adenosine had a role in the anti-ischemic effects of the extracts. However, this hypothesis needs to be more investigated.

## Conclusion

The results of this study showed the aqueous and the ethanolic extracts of *S. leriifolia *radix had protective effects against muscle ischemic injury and significantly decreased the lipid peroxide level in rat muscle following peripheral ischemia- reperfusion damages. The present study showed *S. leriifolia *extracts have protective effect on ischemia reperfusion injury-induced oxidative stress in rats muscle that at least partly due to antioxidant properties of *S. leriifolia *extract.

## Pre-publication history

The pre-publication history for this paper can be accessed here:


